# A Prospective Cohort Study of Bedroom Warming With a Heating System and Its Association With Common Infectious Diseases in Children During Winter in Japan

**DOI:** 10.2188/jea.JE20190312

**Published:** 2021-03-05

**Authors:** Fuyu Miyake, Chimed-Ochir Odgerel, Yuko Mine, Tatsuhiko Kubo, Toshiharu Ikaga, Yoshihisa Fujino

**Affiliations:** 1 Department of Environmental Epidemiology, Institute of Industrial Ecological Sciences, University of Occupational and Environmental Health, Japan, Kitakyushu, Japan; 2 Department of Preventive Medicine and Community Health, School of Medicine, University of Occupational and Environmental Health, Japan, Kitakyushu, Japan; 3 Department of Public Health and Health Policy, Graduate School of Biomedical and Health Sciences, Hiroshima University, Hiroshima, Japan; 4 Department of System Design Engineering, Faculty of Science and Technology, Keio University, Yokohama, Japan

**Keywords:** children, cold temperature, common cold, housing, influenza

## Abstract

**Background:**

Customarily, bedrooms in Japan are left unheated. Although several studies have reported that the use of a heating system has positive outcomes on respiratory infection and asthma, the preventive effect of heating systems against infectious diseases in children is not well known.

**Methods:**

We conducted a cohort study using two questionnaire surveys, one before the winter season in November, 2018 and the second after winter in March, 2019. Participants were 155 children who did not use a heating system in the bedroom and 156 children who did.

**Results:**

Having a heated bedroom with a heating system was associated with decreased odds for the frequency of cold (≥3 times) (adjust odds ratio [AOR] 0.35; 95% confidence interval [CI], 0.19–0.65), duration of fever (≥3 days) (AOR 0.38; 95% CI, 0.22–0.66), duration of medicine for a cold (≥3 days) (AOR 0.91; 95% CI, 0.87–0.95), hospital visit due to cold (≥3 days) (AOR 0.54; 95% CI, 0.31–0.94), absence from school or nursery (≥3 days) (AOR 0.43; 95% CI, 0.27–0.70), influenza infection (AOR 0.43; 95% CI, 0.26–0.71), and gastroenteritis (AOR 0.39; 95% CI, 0.21–0.72). Influenza vaccination reduced the odds of influenza infection (AOR 0.36; 95% CI, 0.22–0.59) and absence from school or nursery (≥3 days) (AOR 0.62; 95% CI, 0.39–0.99).

**Conclusion:**

This study implies that the heating of bedrooms may have a preventive effect against infections among children. Broader dissemination of this knowledge in Japan will require cultural change through public health awareness.

## INTRODUCTION

The Infectious Disease Weekly Report Japan reports that the most common pathogens during the winter season in Japan are viruses, such as influenza virus (IFV), respiratory syncytial virus (RSV), rotavirus, and norovirus.^1^ During winter seasons with low humidity, the specific gravity of viruses becomes lighter, facilitating their air-borne spread. In addition, under conditions of low temperature and low humidity, viruses remain viable for a longer time and remain more stable.^2^ The decrease in body temperature in winter leads to lower metabolism and the suppression of immune responses.^3^ General methods for prevention of common infections include vaccination, washing hands and gargling. Some recent research revealed that a warm indoor temperature is also effective in preventing infectious diseases.^4^^,^^5^ However, the effect of bedroom heating on common infections in children has not been fully elucidated.

IFV and RSV are the primary causes of respiratory infections, such as cough and rhinorrhea, while rotavirus and norovirus are the main causes of gastroenteritis. IFV is a major pathogen in children with acute lower respiratory infection and a substantial burden on health services worldwide.^6^ The best available preventive measure to prevent influenza illness is vaccination.^7^ Other interventions effective in preventing the common cold are physical interventions, such as the use of handwashing and alcohol-based hand disinfectant, and gloves, masks, and gargling.^8^

Rotavirus and norovirus are the most common causes of severe gastroenteritis in infants and young children worldwide.^9^ The frequency of rotavirus infection appears related not to humidity but to temperature. Rotavirus infection is reported to appear when the mean temperature of any 10-day period decreases to <5°C (November or December), to peak at <0°C (January and February), and to wane when it becomes >20°C (June and July) in northern Japan.^10^ Regarding human norovirus infection, norovirus epidemics appear to be associated with a low temperature between −6.6 and 20°C and relative humidity between 10% and 66%.^11^ Both rotavirus and norovirus infection are associated with low temperature.

In recent years, several studies have reported positive outcomes with the use of heating systems, including decreases in absence from school, respiratory infection, and asthma.^4^^,^^5^ In addition, Public Health England (PHE) has established a recommended temperature for indoor environments during the winter season to prevent housing-related health hazards, namely a room temperature of 18°C or more in both the day- and night-time to prevent negative impacts on health.^4^

In Japan, evidence on the health impact of housing is insufficient, and no recommendation for temperature during sleeping time has yet been established.

Historically, warming of bedrooms is not customary in Japan. A recent nationwide survey reported that living room temperature was lower than 18°C for approximately 60% of the study population, and bedroom temperature was lower than 18°C for around 90%.^12^

A recent systematic review^13^ regarding minimum home temperature thresholds for health in winter indicated that an increase in indoor temperature using a heating system has statistically significant beneficial effects on respiratory health in children. However, the specific effect of bedroom temperature remains unknown. According to the cold weather plan for England,^4^ maintaining the 18°C threshold overnight may be less important for healthy people if they have sufficient bedding and clothing and use thermal blankets or heating aids, as appropriate. In this study, we investigated whether the use of a heating system in the bedroom is effective in preventing infectious diseases among children.

## METHODS

We conducted a prospective cohort study from September 2018 to March 2019. For this cohort study, we recruited children aged under 12 years of 1,333 employees of a Japanese company located in Kyushu, a relatively warm area of Japan, which had an average monthly winter temperatures in December 2018, January 2019, February 2019, and March 2019 of 10.2°C, 8.0°C, 9.4°C, and 11.9°C, respectively. Information from 400 children was collected through a baseline questionnaire administered in September 2018. The follow-up survey was conducted in March 2019 and completed by 336 respondents/children (follow-up rate, 84%). We excluded those who had influenza at baseline (*n* = 6) and those who had never used a heating system in the living room (*n* = 19). Finally, a total of 311 children were analyzed.

For the baseline survey, we asked parents about their children’s age, sex, influenza vaccination status, use of a heating system in the children’s bedroom (‘Do you use heating equipment in your children’s bedroom in winter?’), and perception of comfort at bedtime, as well as whether their children had caught a cold, used cold medicine, had a fever, visited the hospital, or were absent from nursery or school due to a cold from September to November, 2018.

In the follow-up survey, the parents were asked how comfortable their children were with regard to bedroom temperature, influenza vaccination status, as well as whether their children had caught a cold, used cold medicine, had a fever, visited the hospital, or were absent from nursery or school due to a cold from December 2018 through February 2019, which is the coldest season in Japan. All participants gave informed consent to participate in the study. The study was approved by the ethics committee of the University of Occupational and Environmental Health, Japan.

### Statistics

The participants were classified into two groups, those who used or did not use a heating system in the bedroom. Descriptive statistics were calculated with respect to demographic indicators and other variables (eg, comfort in the bedroom at bedtime, past history of respiratory problems, and their downstream consequences from September to November 2018). Between-group differences were analyzed using the chi square test. Relationship between the use of a heating system in the bedroom and influenza vaccination, and the likelihood of catching a cold, having a fever, using medicine for a cold, visiting the hospital, or being absent from nursery or school due to a cold, influenza infections, and gastroenteritis were identified using logistic regression. Adjustment was made for confounding factors, including age, sex, influenza vaccination at any time in the year, and prior respiratory problems, and adjusted odds ratios were calculated. Significance was set at the 5% level (*P* < 0.05). STATA Version 15 (StataCorp, College Station, TX, USA) was used for all analyses.

## RESULTS

We studied a total of 311 children, of 156 children did not use a heating system in the bedroom, while 155 children did use a heating system. Table 1 compares the two categories of heating system use by demographic characteristics, vaccination status, perception of comfort at bedtime, as well as whether the child had caught a cold three or more times during the study period from September through November 2018, used cold medicine on 3 or more days, had a fever for 3 or more days, visited a hospital or clinic three or more times, or was absent from nursery or school due to a cold for 3 or more days. Among those who used a heater, 29% of respondents replied that they felt cold, versus 54% of those who did not use a heater (Table 1). Among those who did not use a heating system in the bedroom, 21%, 11%, 34%, 16%, and 12% had caught a cold, had a fever, used medicine for a cold, visited a hospital or clinic, and were absent from nursery or school due to a cold, respectively, from September through November 2018 (Table 1).

**Table 1.** Characteristics of subjects

	Heating system in the bedroom	

	not used	used
	(*n* = 155)	(*n* = 156)
Age, years, %		
0–2	12	23
3–5	25	23
6–12	64	55

Sex, male, %	52	61
Influenza vaccination, %	59	67

Subjective assessment of temperature in the bedroom at bedtime, %		
Comfortable	46	71
Cold	54	29

Past history between September and November prior to follow-up, %		
A cold for ≥3 days	21	13
Fever for ≥3 days	11	12
Medication for ≥3 days	34	29
Hospital visit ≥3 times	16	15
Absent from school or nursery ≥3 days	12	10

Table 2 compares the two categories of heater use or non-use with regard to whether the participant experienced cold or fever, took cold medication, had influenza infection, visited a hospital or clinic, or were absent for cold symptoms during the follow-up period.

**Table 2.** Disease occurrences during the follow-up period (from December through February)

	Heating system in the bedroom		*P*

	not used	used	
	(*n* = 155)	(*n* = 156)	
Cold, %			0.160
none	26	33	
1–2 times	59	57	
≥3 times	15	10	

Fever, %			0.061
none	40	50	
1–2 days	39	38	
≥3 days	21	12	

Medication due to a cold, %			0.266
none	32	40	
1–2 days	29	24	
≥3 days	39	35	

Hospital visit due to a cold, %			0.438
none	37	43	
1–2 times	45	44	
≥3 times	18	13	

Absent from school or nursery due to a cold, %			0.129
none	55	64	
1–2 days	19	19	
≥3 days	26	17	

Influenza, %			0.305
none	79	84	
yes	21	16	

Gastroenteritis			0.297
	86	90	
	14	10	

The percentage of respondents who reported catching a cold (*P* = 0.160), having a fever due to cold (*P* = 0.061), using medicine to treat a cold (*P* = 0.266), visiting the hospital for cold symptoms (*P* = 0.438), taking sick leave for cold symptoms (*P* = 0.129), influenza infection (*P* = 0.305), and gastroenteritis (*P* = 0.297) did not significantly differ among those who did not and did use a heater.

Table 3 shows the relationship between the use of a heating system in the bedroom and the likelihood of catching a cold, visiting a hospital or clinic three or more times, having a fever, taking flu medicine, being absent from nursery and school due to a cold for 3 or more days, and the occurrence of influenza and gastroenteritis. Compared to participants who did not, participants who did use a heater had significantly lower odds of catching cold three or more times (AOR 0.35; 95% CI, 0.19–0.65, *P* = 0.001), having a fever due to a cold for 3 or more days (AOR 0.38; 95% CI, 0.22–0.66, *P* = 0.001), using medicine due to a cold for 3 or more days (AOR 0.91; 95% CI, 0.87–0.95, *P* < 0.001), visiting a hospital or clinic for cold symptoms three or more times (AOR 0.54; 95% CI, 0.31–0.94, *P* = 0.030), being absent from school or nursery due to a cold for 3 or more days (AOR 0.43; 95% CI, 0.27–0.70, *P* = 0.001), being infected with influenza (AOR 0.43; 95% CI, 0.26–0.71, *P* = 0.001), and having diarrhea with vomiting (AOR 0.39; 95% CI, 0.21–0.72, *P* = 0.002). The AORs shown above were adjusted for age and sex.

**Table 3.** Association between use of a heating system in the bedroom and disease occurrence during the follow-up period (from December through February)

	A cold ≥3 times				Fever ≥3 days				Medication due to a cold ≥3 days				Hospital visit due to a cold ≥3 times				Absent from school or nursery due to a cold ≥3 days				Influenza				Gastroenteritis			
						
	OR	95% CI		*P*	OR	95% CI		*P*	OR	95% CI		*P*	OR	95% CI		*P*	OR	95% CI		*P*	OR	95% CI		*P*	OR	95% CI		*P*
Heating system in bedroom																												
no	reference				reference				reference				reference				reference				reference				reference			
yes^a^	0.35	0.19	0.65	0.001	0.38	0.22	0.66	0.001	0.91	0.87	0.95	<0.001	0.54	0.31	0.94	0.030	0.43	0.27	0.70	0.001	0.43	0.26	0.71	0.001	0.39	0.21	0.72	0.002

Heating system in bedroom																												
no	reference				reference				reference				reference				reference				reference				reference			
yes^b^	0.23	0.10	0.49	<0.001	0.27	0.14	0.52	<0.001	0.69	0.42	1.12	0.129	0.28	0.14	0.57	<0.001	0.39	0.23	0.67	0.001	0.55	0.32	0.94	0.028	0.35	0.18	0.69	0.002

Age <6 years																												
Heating system in bedroom																												
no	reference				reference				reference				reference				reference				reference				reference			
yes^b^	0.22	0.08	0.56	0.001	0.17	0.07	0.45	<0.001	0.43	0.19	0.97	0.041	0.20	0.08	0.49	<0.001	0.36	0.15	0.88	0.025	0.29	0.12	0.74	0.009	0.13	0.05	0.36	<0.001

Age ≥6 years																												
Heating system in bedroom																												
no	reference				reference				reference				reference				reference				reference				reference			
yes^b^	0.25	0.04	1.55	0.137	0.41	0.14	1.19	0.101	0.98	0.49	1.96	0.957	0.51	0.13	1.97	0.332	0.61	0.28	1.35	0.224	1.19	0.55	2.59	0.661	0.92	0.30	2.83	0.881

CI, confidence interval; OR, odds ratio.

^a^Odds ratios were adjusted for age and sex.

^b^Odds ratios were adjusted for age, sex, influenza vaccination, and prior respiratory problems.

In addition, we examined the interaction effect of age and sex on the association between a heating system in the bedroom and health outcomes. Sex did not show significant interaction effect on the association. However, we found a strong interaction effect of age on the association, so we further conducted subgroup analyses stratified by age group. The associations of a heating system in a bedroom and health outcomes were greater in the group aged under 6 years than the group aged 6 years and over in all outcomes measured (Table 3).

Table 4 shows the relationship between flu vaccination and the likelihood of catching a cold, visiting a hospital or clinic three or more times, having a fever, taking medicine for a cold, being absent from nursery or school due to a cold for 3 or more days, and the occurrence of influenza and gastroenteritis. Compared to those who did not undergo influenza vaccination, those who were vaccinated had significantly lower odds of taking leave from school or nursery (AOR 0.53; 95% CI, 0.30–0.95, *P* = 0.034) and being infected with influenza (AOR 0.44; 95% CI, 0.25–0.79, *P* = 0.005). The AORs shown above were adjusted for age, sex, flu vaccination, prior respiratory problems, and use of bedroom heating. No significant relationships were found between flu vaccination and these downstream consequences (catching a cold three or more times, having a fever, taking medicine to treat cold symptoms, being absent from school or nursery due to cold for 3 or more days, and having diarrhea with vomiting).

**Table 4.** Association between vaccination of influenza and disease occurrence during the follow-up period (from December through February)

	A cold ≥3 times				Fever ≥3 days				Medication due to a cold ≥3 days				Hospital visit due to a cold ≥3 times				Absent from school or nursery due to a cold ≥3 days				Influenza				Gastroenteritis			
						
	OR	95% CI		*P*	OR	95% CI		*P*	OR	95% CI		*P*	OR	95% CI		*P*	OR	95% CI		*P*	OR	95% CI		*P*	OR	95% CI		*P*
Influenza vaccination																												
no	reference				reference				reference				reference				reference				reference				reference			
yes^a^	0.76	0.43	1.37	0.366	0.75	0.45	1.26	0.277	1.24	0.81	1.91	0.317	1.39	0.79	2.44	0.249	0.62	0.39	0.99	0.047	0.36	0.22	0.59	<0.001	0.80	0.45	1.43	0.460

Influenza vaccination																												
no	reference				reference				reference				reference				reference				reference				reference			
yes^b^	0.68	0.31	1.49	0.338	0.79	0.41	1.54	0.493	0.87	0.50	1.49	0.610	1.60	0.77	3.34	0.209	0.53	0.30	0.95	0.034	0.44	0.25	0.79	0.005	1.15	0.56	2.37	0.703

CI, confidence interval; OR, odds ratio.

^a^Odds ratios were adjusted for age and sex.

^b^Odds ratios were adjusted for age, sex, influenza vaccination, prior respiratory problems, and use of bedroom heating.

## DISCUSSION

This study suggests that using a heater in the bedroom at bedtime is strongly associated with good health in children. We found that the chance of catching a cold three or more times, having a fever due to a cold for 3 or more days, using medicine for a cold for 3 or more days, visiting the hospital or clinic for cold symptoms three or more times, being absent from school or nursery due to a cold for 3 or more days, being infected with influenza, and having diarrhea with vomiting among children decreased with the use of a heater in the bedroom at bedtime. For example, compared to children who slept in a bedroom with no heater, those who used a heater had significantly lower odds of catching a cold three or more times during the study period (AOR 0.35; 95% CI, 0.19–0.65; *P* = 0.001).

This result is closely consistent with those of many previous studies. For example, Braubach et al reported that in children,^14^ the excess winter health burden is mostly due to respiratory disease, while excess winter deaths due to cold housing has been estimated at 38,200 per year (12.8/100,000) in 11 selected European countries.^15^

Other researchers have also shown that colder indoor temperatures increased respiratory morbidity.^5^^,^^16^ Cold housing causes poor respiratory health^17^^–^^20^ among both adults and children. However, adults with chronic respiratory diseases had a better health status with more hours of indoor temperature above 21°C. Mu et al^21^ also revealed that respiratory problems among adult COPD patients were reduced with an indoor temperature at 18.2°C. In contrast, a case-control study in children with and without upper respiratory tract infections showed no consistent associations with indoor temperature.^22^ This may reflect the fact that all children are vulnerable to cold indoor temperature regardless of the presence of any ill condition. In addition, a recent systematic review in a broad target population, which included vulnerable groups, suggested that there is a lack of epidemiologic studies examining the effect of room temperature on health outcomes.^23^ However, some studies involving asthmatic children found that every 1°C increase in room temperature above the threshold of 9°C was associated with a small but significant increase in lung function.

Some parents believe that vaccination is a way to prevent any respiratory infection. However, a vaccine is a biological preparation that improves immunity to a particular infection, but not to all respiratory infections. Our study results showed that, compared with those who were not vaccinated for influenza, vaccinated children had much lower odds of being infected with influenza; however, no relationship was seen between vaccination and decreased chance of catching cold. This may support the accuracy of our questionnaire in measuring what it claimed to measure. In addition, our study suggests that using a heating system in the bedroom has significant benefit in preventing respiratory infections in children regardless of vaccination. This is supported by a previous study in animals, which showed that both relative humidity and temperature are important in suppressing influenza infection and that an indoor temperature of more than 20°C appears favorable.^3^

Our present study showed that children who used a heating system in their bedroom had a lower chance of having diarrhea with vomiting (AOR 0.39; 95% CI, 0.21–0.72; *P* = 0.002) than those who did not use a heating system. The major pathogens of these symptoms are viruses, such as noroviruses and rotaviruses, which spread during the winter season. Some reports have indicated both norovirus and rotavirus become active at less than 20°C.^10^^,^^11^ Regarding infection route, while cold symptoms are generally spread by droplet infection, diarrhea with vomiting is spread by contact infection. Considering the route of infection, even when no individual infected with a virus that causes diarrhea with vomiting is present, the possibility of developing gastroenteritis remains if the virus remains in the bedroom below 20°C.

Many houses in Japan are not well insulated, and residential energy consumption is low compared to American and European countries, with Japan using only one-quarter of the heating energy used by Western countries.^24^ In Japan, it is standard practice to heat only the living room rather than the entire apartment or house, as is seen in Europe and America. Heating the bedroom is uncommon, especially in the Kyushu region. As a result, the average bedroom temperature in Japan is lower than in other countries.^12^^,^^25^ Of note, it is below the minimum temperature recommended by the World Health Organization^15^ and the United Kingdom government.^26^ “Warming up at bedtime” in Japanese culture means to sleep wearing warm nightwear and covered with a thick blanket. While adults can generally manage a feeling of coldness during sleep by covering themselves with a blanket, young children are unlikely to cover themselves when they feel cold. Heating the bedroom during sleep is, therefore, crucial. Some studies in asthmatic children have shown that bedroom exposure has a stronger association with lung function than living room exposure.^5^

Our study results are also supported by other pathophysiological evidence. Most available evidence from laboratory and clinical studies suggests that inhalation of cold air, cooling of the body surface, and the cold stress induced by lowering the core body temperature cause pathophysiological responses (eg, vasoconstriction in the respiratory tract mucosa and suppression of immune responses) that increase susceptibility to infection.^27^ In addition, many researchers have studied the influence of cold exposure on immune function.^28^ The data, obtained mainly in small mammals, suggest that the acute effect of severe chilling is a suppression of several cellular and humoral components of the immune response.

Deprived and vulnerable households are more likely to live in energy-inefficient houses and less likely to have the resources or resilience to deal with the negative impact of a cold home and insufficient income.^29^ Although the workers whose children were included in the present study did not fall into the category of “deprived or vulnerable”, half nevertheless reported that they did not use a heater in the bedroom. In fact, the parents of the subjects in the present study are regular employees of a company which provides an average annual salary of more than $55,000, which is much higher than the approximately $36,000 average annual income in Japan. Thus, it is critically important that parents be educated on the need to maintain a warm indoor environment.

There were some inconsistencies between the results of Table 2 and Table 3. Logistic regression showed the significant associations of a heating system in the bedroom and health outcomes, whereas the Chi square test showed no difference in the frequencies of health outcomes between the groups. We consider that this is due to the fact that the logistic regression accounted for age, sex, and other covariates. In addition, outcome values in the logistic regression were defined as a higher frequency group of outcomes, such as a three times and higher outcome of catching a cold. The health outcomes in this study are common diseases among children in winter, and if a cold indoor temperature confers an excess risk of disease, it is reasonable to assume an increase in the per-person number of cases of a disease, rather than in the experience of having a disease (ie, whether a child has a cold more than once).

We consider that the present study has several limitations. First, we did not measure actual bedroom temperatures or the frequency of nighttime bedroom heating. Rather, we considered that the bedroom would be warm if a heater were used and vice-versa. This means that we were unable to examine whether increasing the bedroom temperature changed the health condition, or suggest a recommended bedroom temperature that appears to support the health of children. We plan to measure bedroom temperatures and evaluate the relationship between a change in room temperature and health status in a future study. Second, we did not consider the effect of humidity, which is also an environmental factor related to infectious disease in winter. Low humidity damages the oral, nasal, and bronchial mucous membranes, which in turn facilitates viral invasion and infectious disease.^3^ Third, we did not collect information on pre-existing conditions, such as low-birth-weight babies or prematurity and congenital diseases, which might have had confounding effects on the results. Fourth, the validity of the questionnaires used in the present study is unknown. We did not investigate the houses in which the subjects lived. However, we speculate that the giving the wrong answer to questions on whether they have a heating system or not would be very unusual. In addition, in terms of the outcomes, we consider that there was little possibility of parents’ providing incorrect answers to questions regarding the number of hospital visits and the number of cold caught over a 3-month period. Even if the parents gave a wrong answer about the number of hospital visits or catching a cold, this might not be biased by usage of a heating system in a bedroom. Any instance of giving the wrong answer would likely result in attenuation of the observed associations between a heating system and outcomes.

However, the study also has two notable strengths. First, the participants were quite homogeneous in terms of their residential region and socioeconomic status, which should have reduced potential confounding related to socioeconomic factors (eg, nutritional status, poverty level, and access to health care). Second, the participants were asked to record on paper the outcome variables (cold, fever, hospital visit, sick leave, and medication use) during the coldest seasons (December 2018 through February 2019) and to complete the questionnaire in March 2019. We consider that this methodology likely attenuated the recall bias that frequently impacts studies involving self-reported measures, to some degree at least.

In conclusion, this study revealed that the use of a heating system in the bedroom has an effect in preventing the onset of colds, flu, and gastroenteritis among children. In addition, heating also reduces absence from school. This study emphasizes the importance of warming the bedroom in winter in Japan, where central heating systems are not common. Ensuring understanding and compliance with this information will require cultural change through public health awareness.
